# Health care providers' attitudes towards termination of pregnancy: A qualitative study in South Africa

**DOI:** 10.1186/1471-2458-9-296

**Published:** 2009-08-18

**Authors:** Jane Harries, Kathryn Stinson, Phyllis Orner

**Affiliations:** 1 Women's Health Research Unit, School of Public Health and Family Medicine, Faculty of Health Sciences, University of Cape Town, Anzio Road, Observatory, 7925, Cape Town, South Africa

## Abstract

**Background:**

Despite changes to the abortion legislation in South Africa in 1996, barriers to women accessing abortion services still exist including provider opposition to abortions and a shortage of trained and willing abortion care providers. The dearth of abortion providers undermines the availability of safe, legal abortion, and has serious implications for women's access to abortion services and health service planning.

In South Africa, little is known about the personal and professional attitudes of individuals who are currently working in abortion service provision. Exploring the factors which determine health care providers' involvement or disengagement in abortion services may facilitate improvement in the planning and provision of future services.

**Methods:**

Qualitative research methods were used to collect data. Thirty four in-depth interviews and one focus group discussion were conducted during 2006 and 2007 with health care providers who were involved in a range of abortion provision in the Western Cape Province, South Africa. Data were analysed using a thematic analysis approach.

**Results:**

Complex patterns of service delivery were prevalent throughout many of the health care facilities, and fragmented levels of service provision operated in order to accommodate health care providers' willingness to be involved in different aspects of abortion provision. Related to this was the need expressed by many providers for dedicated, stand-alone abortion clinics thereby creating a more supportive environment for both clients and providers. Almost all providers were concerned about the numerous difficulties women faced in seeking an abortion and their general quality of care. An overriding concern was poor pre and post abortion counselling including contraceptive counselling and provision.

**Conclusion:**

This is the first known qualitative study undertaken in South Africa exploring providers' attitudes towards abortion and adds to the body of information addressing the barriers to safe abortion services. In order to sustain a pool of abortion providers, programmes which both attract prospective abortion providers, and retain existing providers, needs to be developed and financial compensation for abortion care providers needs to be considered.

## Background

Providing greater access to safe abortion reduces the public health burden of unsafe abortion, which in 2004 was estimated at 68,000 deaths and five million permanent or temporary disabilities per annum, primarily in developing countries [1]. In countries where legislation permits termination of pregnancy (TOP), access to safe induced abortion may be restricted due to limited numbers of trained health care providers [2]. To improve access to safe abortion and conserve scarce health resources, some countries, including South Africa, have trained mid-level providers (MLP), i.e. health care providers who are not doctors, such as midwives and registered nurses, to perform first trimester abortions [3].

In South Africa, the Choice on Termination of Pregnancy Act (CTOP) (No. 92 of 1996) promotes a woman's reproductive right and choice to have an early, safe and legal abortion. The CTOP Act allows for first trimester abortions (up to 12 weeks gestation) to be performed by mid-level providers comprising trained professional nurses and midwives, whereas second trimester abortions are provided by doctors. As a result of this abortion legislation, abortion related morbidity and mortality have decreased significantly by 90% [4]. However, despite liberal abortion reform laws there are still major barriers to women accessing abortion services. These include provider opposition, stigma associated with abortion, poor knowledge of abortion legislation, a lack of providers trained to perform abortions and facilities designated to provide abortion services particularly in the rural areas [5-10].

Abortion is a time-restricted health service. The current legislation provides for abortion on request up to and including 12 weeks of gestation. In cases of socio-economic hardship, rape, incest and for reasons related to the health of the pregnant woman or foetus, terminations can be performed up to 20 weeks of gestation. From 20 weeks onward terminations are available only under very limited circumstances.

The CTOP Act makes allowance for a health care provider's right to conscientious objection. This right is supported by the constitutional rights of all South Africans to freedom of thought, belief and opinion. A health care provider may refuse to perform an abortion, however, they are obliged to inform a woman of her reproductive right to choose an abortion according to the Act, and to refer her to another provider or facility. Ethical guidelines have been drafted by the South African Nursing Council with regards to conscientious objection and abortion provision. A nurse refusing to participate in the act of performing a TOP must lodge in writing to their employer his or her refusal to carry out an abortion. Irrespective of conscientious objection, a nurse must provide nursing care including assistance with activities of daily living, emotional support, prescribed medications and comfort and pain relief measures.

In recent years the number of abortions performed nationally and in each of the provinces, including the Western Cape has increased substantially, indicating increased availability and accessibility to abortion services [11]. Despite this manifold increase in demand and utilization, challenges exist in the further expansion of services, particularly through trained nurse or midwife service provision up to 12 weeks gestation [12]. The shortage of health care providers who are willing or trained to perform abortions undermines the provisions of the CTOP Act, by limiting the availability of safe, legal abortion, and has serious implications for women's access to abortion services and health service planning.

There has been little research to date on health providers' attitudes towards abortion in South Africa [5,9]. Studies elsewhere, including countries where abortion is highly restricted, have found that various factors shape health professionals' attitudes towards induced abortion [13]. Religious beliefs, the reasons for seeking an abortion such as rape or incest, and gestational age were all found to affect attitudes and willingness towards abortion provision [13-15]. In South Africa, little is known about the personal and professional attitudes of individuals who are currently working in abortion service provision. Exploring the factors which determine health care providers' involvement or disengagement in services may facilitate improvement in the planning and provision of future services.

This paper reports on results from a qualitative study that explored knowledge, attitudes and opinions of health service providers who are likely to play a critical role in determining access to and the quality of these services.

## Methods

### Study sites

The study was conducted between July 2006 and October 2007 across 3 public sector primary health care facilities; 8 hospitals; 4 non-governmental organization (NGO) facilities, 3 of which provided abortions and 1 providing pregnancy counselling; and 2 health services linked to secondary and tertiary educational institutions. Research sites were based within the greater Cape Town area and three outlying areas within the Western Cape Province, South Africa. Facilities provided a range of representative services from pre abortion counselling and referral, to the provision of first and second trimester abortions, post abortion counselling and contraceptive services. The study was based in the Western Cape for logistical and funding reasons.

Abortions at public sector facilities were available free of charge, while NGO facilities offered a mix of free and fee-related services. Facilities that offered first trimester abortions were either provided by mid-level providers who had been trained in manual vacuum aspiration (MVA) techniques or by doctors. Facilities that provided both first and second trimester abortions employed registered nurse midwives for terminations up to 12 weeks, and doctors for second trimester abortions. Some doctors who provided second trimester abortions formed part of a "roving team" of providers who rotated between public sector facilities in the study sites. Most second trimester abortions were performed using the dilation and evacuation (D & E) method. Misoprostol (Cytotec^®^) was administered to all abortion patients for cervical priming prior to the MVA and D & E procedure. Medical abortion for first trimester abortions is not currently available in the public sector. The medication method of abortion for second trimester abortions using misoprostol-alone which requires a hospital admission of several days was used in some tertiary hospitals. The Western Cape is the only province in South Africa where D & E for second trimester abortions is currently offered on a limited scale in the public sector. In most other provinces in South Africa within the public sector, the medication method of abortion using misoprostol-alone is the preferred method for second trimester abortions.

### Study respondents

A total of 34 in-depth interviews and one focus group discussion (comprising 4 counsellors) were conducted with health care providers who were involved in a range of aspects of abortion service provision (see Table 1). The majority of respondents were female (89%) and the median number of years of experience in abortion services was 7 years (range 0–30). Participants were selected through purposive sampling. Due to the paucity of providers, snow ball techniques were used to identify both providers and non-providers for the study.

**Table 1:** Background variables associated with TOP service provision

Study sites	Primary Health Care facility	3
		
	Hospital (Level 1, 2 or 3)	8
		
	Non-governmental organization	4
	Other	1
	Total	16
		
Training status	Providing service – Trained	16
	Providing service – Not trained	0
	Providing service – Health care manager	1
	Providing service – Total	17
		
	NOT Providing service – Trained	1
	NOT Providing service – Not trained	17
	NOT Providing service – Health care manager	3
	NOT Providing service – Total	21
		
Service provider category	Counsellor	7
	Enrolled Nurse	2
	Registered nurse	3
	Nurse-midwife	17
	Doctor	6
	Management	3
		
Median number of years worked in TOP services (range)		7 (0–30)
		
Sex of provider	Male	4
	Female	34
		
Religious affiliation	Christian	19
	Other	1
	Not specified	18

The sample represented a range of health care providers who varied by professional category (including doctors, registered nurses and midwives) and type of provider. These included providers who were trained to perform abortions and were providing abortions; providers trained in abortion services but were not providing abortions, and providers who were not trained in abortion procedures. Other respondents included nurses and counsellors who were involved in pre and post abortion referral and counselling, and health care managers in facilities providing both abortion and/or reproductive health care services.

### Study design

Qualitative in-depth interviews were conducted among health care providers and health care managers who were working in facilities that provided abortion services in the public, private and NGO sectors. Owing to the sensitivity of the subject matter, and respect for privacy of participants, individual interviews were deemed the most appropriate method for data collection. A single focus group discussion was held with four participants on their request, as they felt more comfortable speaking in a group rather than on an individual one on one basis. The interview guide was adapted accordingly to facilitate discussion.

Interview guides were semi-structured, open-ended, and made use of probes. Socio-demographic data were collected prior to the interview, and included gender, religious affiliation, training and qualifications, category of provider and years of experience as a provider. A pilot study was conducted to check for appropriateness and understanding, and revisions were made to improve the clarity and flow of the instrument. Interviewers, who had experience in qualitative research methods, conducted the interviews in English. Interviews were approximately an hour in duration and were held in a private setting.

Interviews were digitally recorded and transcribed verbatim. All participants provided written informed consent, and confidentiality and anonymity were ensured. Ethical approval was obtained from the Research Ethics Committee, University of Cape Town and the World Health Organization Research Ethics Review Committee. Approval to conduct the study was obtained from the Western Cape Provincial Department of Health and from the NGO facilities.

### Data analysis

Data were analysed using a thematic analysis approach. Initial categories for analysing data were drawn from the interview guide and themes and patterns emerged after reviewing the data. Key themes to emerge were: reasons providers were not willing to provide abortions including individual and health service related barriers, how providers defined or conceptualised abortion, knowledge and understanding of the TOP legislation, and how reasons for seeking an abortion impacted on providers' decisions to be involved in abortion provision.

The computer software package ATLAS ti 5.2 was used to facilitate sorting and data management (Scientific Software Developments, 1998–2008). Members of the research team developed and refined the codes using the key issues probed. The transcripts were coded by the research team and then cross checked for coder variation. The data were then reviewed for major trends and crosscutting themes were identified. Issues for further exploration were prioritised for final analysis. No coding discrepancies were encountered.

## Results

Complex patterns of service delivery were prevalent throughout many of the health care facilities, and fragmented levels of service provision seemed to operate in order to accommodate health care providers' willingness to be involved in different aspects of abortion provision. Some providers provided abortions and some assisted with the procedure and/or provided pre and post abortion counselling. Others restricted their involvement to tasks solely relating to pre abortion care, such as performing ultrasounds to determine gestational age and referral to a designated abortion facility.

### Knowledge of TOP legislation

Knowledge of the current abortion legislation and the right to conscientious objection (refusal on religious grounds to performing an abortion but the requirement to refer to another facility or provider) varied amongst both providers and non-providers.

Providers who were performing abortions were aware of the CTOP Act, however, some providers who were supportive of a woman's right to choose were not all that familiar with the legislation. Non-providers who were opposed to abortion were unclear about the conditions under which a woman could request an abortion. There was confusion regarding gestational age requirements and who was legally able to perform an abortion. When asked about the legislation, a non-provider's response was:

*I'm not too familiar with the Act at all, just the little snippets that I picked up from the previous nurse who worked here. She used to quote the Act. I do know vaguely, I think that they do have to still see a doctor, who will then decide whether that person is entitled to have the abortion*.

#### Barriers to service provision

##### Conscientious objection

An ad hoc interpretation of the right to conscientious objection was reported by many respondents, who felt that it often interfered with abortion service provision.

Confusion and uncertainty with regards to conscientious objection was reported by both providers and non-providers. There was a general lack of understanding concerning the circumstances in which health care providers were entitled to invoke their right to refuse to provide, or even assist in abortion services. According to respondents, it was evident that health services lacked the necessary regulatory structures to deal with conscientious objection among health care providers. Furthermore, there seemed to be very little recognition or support from health service managers regarding effects of conscientious objection on service provision. Many providers reported that staff including non nursing staff such as cleaners and administrative personnel refused to assist or provide basic nursing care to abortion clients. A nurse provider at a public sector designated abortion facility explained how access to care had been blocked by an admissions clerk.

You'll see the way they'll treat patients who come with the letter...because they must have a referral letter. Once they open that letter and find it's a TOP, they will just throw that letter away and if it's somebody who asked them to open a folder for my clients as if it's she who's actually doing the TOP. Or it just might happen, if they come early at half past 6 in the morning, they'll only be admitted at 11 o'clock. The whole day they are sitting there in the corner, they didn't have somebody to take care of them because they've come to do this obscene something that cannot be heard of, but now I've got a dedicated clerk, now everything is running smoothly again..

Many designated public sector facilities did not have providers who were prepared to either perform abortions or to assist those performing abortions. Abortion services were often not provided due to "pro-life doctors not wanting to do anything about abortions", resulting in a roving team of providers from the private sector providing the services. The impact of conscientious objection on service provision included all aspects of the abortion process from refusing to prescribe or administer necessary medications to refusing to assist in the operating room or provide abortions.

I'm the only one who's happy to do it [abortions], because the other nurses refused to give even the tablets [misoprostol] for the girls. She helps me in theatre, making the packs and assisting me with the book work but she refuses to give the tablets. She said she's not going to involve herself with this.

However, some providers mentioned that those who refused to be involved with abortion care would assist for financial compensation.

##### Empowerment and shifting role of mid-level provider

The impact of the shifting role of the mid-level provider as a result of policy change post 1996 was accompanied by varying degrees of willingness of staff to become involved in abortions. As mid-level providers had never previously provided abortions, some working in family planning found the adjustment to a "new environment" challenging as many colleagues were not willing to change their attitudes.

*I mean it was very difficult for me because it was a very antagonistic position to be in – everybody wanted to see what we were doing but nobody was willing to help the patients who were having abortions.*.

However, for some mid-level providers the new context of being able to perform first trimester abortions was seen as "empowering" and an opportunity to broaden their skills base and provide a comprehensive service to women with unplanned pregnancies.

A nurse provider sought to combine operating room experience with opportunities for abortion training which was offered after the CTOP Act was passed. In this new context, being an abortion provider and being able to perform first trimester abortions was seen as empowering particularly for mid-level providers.

As a nurse-midwife stated:

TOPs are a very empowering sort of thing for providers, nurse providers, ...you become more confident, you do a different function that is not in your normal role, people now look at you differently.

#### Influencing factors in abortion provision

##### Personal reasons

Reasons for involvement in abortion provision were often tempered by indirect or direct personal experiences. For some, provision was part of a natural career trajectory, whereas for others, involvement was linked to prior exposure to mortality and morbidity associated with illegal "backstreet" abortions and the recognition of a dearth of providers willing to provide abortion services.

A nurse-midwife underscored her need to be involved in services from their inception thus:

I think having nursed patients who came in with septic abortions, and who were quite ill and distressed...subsequently, even though there was a big space of time between nursing them and eventually coming to work in TOP, that was one of my motivating reasons. And also the fact that it was a new Act that came in – legislation that was implemented – and being part of that, was very important for me, because it all had to do with women's empowerment, you see.

Other health care providers suggested that involvement in abortion service provision was a vocation requiring passion and commitment. One provider emphasised:

I think that people must choose to be in that situation, because some people are very anti abortion and you can't force somebody that is totally anti abortion, to go and work with somebody who is having an abortion.

##### Moral reasons

Abortion as a moral choice and how it influenced health care providers' degree of involvement in services was framed in different ways. Some providers were vehement in their dislike of abortion care, whereas others were prepared to restrict their involvement to pre and post abortion counselling or basic nursing duties, and were not willing to provide direct abortion care including performing abortions. As a mid-level provider stated:

I don't want to come to do TOPs ... I would just hate it, hate it, hate it, it's not my choice ...I want to enjoy my work.

While some non-providers working in the services did not "like" abortion, they emphasised that they did not feel that it was "wrong", or that it was their place to "judge a client".

Providers not directly involved in abortion services who based their objection to abortion on moral grounds, tended to have different thresholds in terms of their willingness to assist in preparing clients for an abortion. For instance, mid-level providers described that they would help with ultrasounds and pre abortion counselling, or they would set surgical trays, but they preferred not to assist in the procedure itself. Some said that they went so far as to absent themselves from the room during the procedure. One non-provider refused to administer misoprostol as she believed it was an abortifacient and explained her reasons thus:

I refused to give the misoprostol because I'm helping it on ... I don't think it's good to take a life, that's my point of view.

Providers who stated that they were "pro-choice" were more likely to talk about a "woman's right to choose". They maintained that a lack of objectivity regarding a woman's right to choose arose from pre judging women as irresponsible without thinking of the long term consequences of an unplanned pregnancy. A nurse provider felt that it was "sinful" to bring children into the world when they were at risk of being neglected and not adequately provided for. She mentioned:

When I speak to anybody about preserving life, I am thinking of the life of this woman. I also think I always bring them back to the fact that abortion – being pregnant – has many options, that women will, or people who are there will say, "what about the life of the unborn?" Now what would the quality of life be if the unborn was born, and it was not born into happy circumstances, and where it could be provided with the basic needs?

##### Religious beliefs

When asked about the role of religion as an influential factor in service provision, most health care providers had experienced colleagues' opposition to abortion on a mix of religious and moral grounds in the working environment. Many abortion providers were regarded as "murderers" and "baby killers" who were expected to "preserve and not take life".

Religious beliefs played a role for some providers in deciding not to be involved in abortion services. As one non-provider explained:

*... I'm Catholic, but I'm also doing family planning, so it doesn't seem to make sense. But I thought I have to take a stand somewhere on something, so that's why when I went into this position I told them I didn't want to do – take part in any of that*. [Referring to abortions]

In contrast, another provider approached the issue differently, stating that she was a practising Catholic but "had made peace" with her decision to provide abortions despite the fact that she had been ostracised by her religious community.

Despite personal or religious beliefs prohibiting TOP involvement, some providers were able to separate personal values from professional conduct. Providers who described themselves as "pro-choice" favoured a "clinical" over an "emotional response" to abortion, viewing abortion care as "part of their job", whereas those opposed to abortion found it difficult to separate their personal feelings from professional conduct.

#### Reasons for seeking an abortion

##### Rape, incest and foetal abnormality

We explored whether the reasons why a woman sought an abortion could influence providers' attitudes towards providing care. Almost all providers perceived an unplanned pregnancy due to rape or incest as different and a legitimate reason to obtain an abortion. The few providers who commented on foetal abnormality suggested that staff generally were more understanding and supportive towards a woman seeking an abortion for what they perceived as a legitimate medical reason. It was assumed that a woman would be more traumatised about giving birth to a baby with a foetal abnormality and therefore deserving of more support, and that this would be forthcoming from staff irrespective of their stance on abortion.

##### Socio-economic

Respondents appeared to be in agreement that many women who sought abortions were motivated by socio-economic hardship. Whether it was due to being too young, having to defer studies, or just being too poor and overwhelmed to have another child, participants responded with sympathy and understanding. Reflecting on a possible miserable life for a woman and her child, many respondents were clear that women should not have an unaffordable baby. It was thus critical not to delay an abortion in these circumstances, as any delay could result in women changing their minds. Comparing their own relatively better circumstances to that of many abortion seekers seemed to elicit a sympathetic response from some providers, including those who personally would have not opted for an abortion.

#### Experiences: abortion services

The effective provision of abortion services seemed to be contingent on the willingness of staff to be involved in provision. Respondents suggested that frequently those who were providing abortion services felt stigmatised. Service providers experienced "burnout" and left the services as "they could not endure the comments or the attitudes of their colleagues". Another provider described feelings of isolation experienced by some nurse providers:

They make it difficult for you. They spread the word in the community...and also isolate you. Where you're supposed to be peers and working hand in hand and you can become extremely unhappy. You'd often find midwifes not providing abortions because they fear the victimisation, being stigmatised, being isolated from their peers, and also within the community itself.

##### First and second trimester abortions

While the importance of providing second trimester services was recognised, attitudes towards it varied, with the majority of providers feeling distinctly uncomfortable about second trimester abortion provision. Some were absolutely opposed to second trimester abortion, while others felt it was a procedure they could come to terms with over time. Non-providers who refused to involve themselves in abortion care were particularly vehement in their opposition to second trimester abortions, and in certain instances refused to prescribe or administer misoprostol for women presenting for second trimester abortions. Gestational age was a key indicator of acceptability. Providers found it more traumatic to deal with a termination performed around 17–20 weeks, than a termination at 14 weeks, because with the latter, one was dealing with an embryonic sac rather than a "formed foetus".

Providers expressed concern regarding the increase in the number of second trimester abortions at facilities. Several respondents attributed this rise to a health systems failure and a scarcity of providers. Others suggested that women were not aware of their rights, or simply did not know where to go to access the service. Some found dealing with clients who requested second trimester abortions frustrating, because they found it difficult to understand why women "waited so long".

#### Contraceptive services

Discussion on contraception was couched in terms of failure – failure of the public health sector to provide effective services and failure on the part of clients to use contraceptives. Often this was followed by reference to women preferring abortion as a means of contraception. A common perception amongst respondents was that contraceptive services in the public health sector were not only preferable to abortion but were essential to the health of women. Yet, there were multiple barriers to this becoming the reality, including little or no contraceptive counselling, limited contraceptive choice and judgemental attitudes particularly towards younger women. Post abortion counselling was difficult to initiate as providers often had to talk to women "on the run" or they were too rushed to provide comprehensive post abortion contraceptive counselling and was easier to continue giving injectable contraceptives.

Some providers remarked that family planning clinics had been replaced by comprehensive health care and staff were not adequately trained in family planning which meant that:

Everybody gets injections and then you...fall pregnant and then you get it again. So it is injection, injection, injection and then the message everywhere is condoms. So when you ask the young girls, "What do you use for contraception?" you know they use condoms, but if you ask them, "Do you use them all the time?" it's invariably no...I would like to see ...nurses going to schools and talking about family planning at school level.

##### "Repeat abortions"

"Repeat abortions" or the possibility that women were using abortion as a contraceptive method appeared to be a major concern for many participants in this study and influenced their decisions around abortion provision and care. A woman who returned for a second or third time was identified as coming for a "repeat abortion" and in turn was perceived as sexually irresponsible. The relationship between failed or no contraception was linked to concerns with the "break down" in current family planning services including inadequate family planning counselling.

...For some people it's just becomes too easy an option to choose because I've seen people coming in for second and third abortions and it's, I mean I see that it's becoming a contraceptive and so that doesn't sit well with me at all.

However, while it was clear that a number of participants felt it was unacceptable that clients would use abortion as a contraceptive method, others attributed the rise in "repeat abortions" to the dearth of family planning services, inadequate contraceptive counselling and difficulties in accessing services.

#### Quality of services

Discussions around current service provision suggested a tremendous concern about quality of care within the public sector health facilities. Providers' concerns centred on problems associated with a general lack of adequate pre and post abortion counselling, punitive staff attitudes towards younger women, overcrowded, overburdened and fragmented services, and difficulties with staff recruitment and retention.

A nurse provider described how often large numbers of women were turned away due to staff shortages and the potential consequences of this.

...There is often not enough staff to provide the services and we are only able to take a certain number of clients a day, women are often too scared to go elsewhere or can't afford transport fare to attend another facility, resulting in them seeking a back street abortion.

Large patient numbers and limited suitable private spaces at designated facilities made it extremely difficult to provide adequate counselling and care. Providers described some of the dilemmas they faced:

Women are hanging around in rooms...waiting and having foetuses between their legs for hours and nobody really cares. But on the other hand, I'm delighted that at least it gets done ... [Doctor providing abortions]

A respondent from an NGO providing reproductive health care services summed up the situation in public sector facilities.

I think the way in which TOPS are done in the government clinics at the moment is really not working because it's not integrated with other services, it is completely overloaded and there's no privacy. It's just not a quality service and they really should be reviewed in its complete sense to really look again at policies of implementation and to make sure that this Act is implemented the way it should be.

However, there were also positive comments about the services particularly within the NGO sector where providers felt they had more time for counselling and appropriate infrastructure to provide optimal services and where people who worked there chose to be involved in abortion provision. This was preferable to many public sector facilities where TOP services had been introduced as part of sexual and reproductive health care services and had not necessarily obtained the "buy-in" from health personnel working there.

##### Values clarification and abortion training

A conflicting and complex picture of the state of abortion training opportunities and training barriers for mid-level providers arose during discussion. Training opportunities for some appeared to constitute training barriers for others. On the one hand, access to training was unproblematic for those who sought it: "They just have to phone the college and they'll get training, whenever there's a training group." On the other hand, training opportunities were described as sporadic and frequently subject to cancellation due to insufficient interest from providers. Staff shortages at health care facilities made it difficult for those who wanted to undergo training to be released as there were often no staff replacements.

Stigma and fear associated with providing or even assisting with abortion services appeared as a serious barrier to accessing training, even when it was on offer. Attending abortion training could be particularly fraught for some providers as training could signify a pro-choice stance, consequently opening oneself up to "finger pointing".

Values clarification workshops were introduced in South Africa shortly after the implementation of the new CTOP Act and were facilitated by international NGOs such as Ipas, in collaboration with local health departments. The aim was to allow health care providers and other key stakeholders to clarify their values and attitudes and engender changes in attitude and behaviour towards women seeking an abortion. When starting a new position in abortion service provision, whether as a provider or a non-provider, attendance of values clarification workshops were not mandatory, and there seemed to be little done to encourage or regulate attendance. There was an overriding sentiment that values clarification workshops had a positive impact on service provision assisting those opposed to abortion "to viewing things differently". Providers suggested that values clarification helped them to define their role as facilitators who guided rather than directed a client. This alleviated the decision-making process. One provider remarked:

TOP is not a nice thing. Values clarification opens up your mind ...After that I don't have to judge that woman because I don't have a right to judge. She's got her own reasons why she's doing this and you have to respect that client as a patient.

#### Possible intervention strategies

Most providers spoke about the need for "dedicated centres for TOPs" or "special abortion clinics" to create a more supportive environment for both clients and providers. Many saw this as a way of dealing with negative staff attitudes and with providers who refused to be involved in abortion care and provision. A nurse provider explored the complexities of providing abortion care in a climate of resistance to abortion stating that:

People who work in this area must be committed and passionate about it. I think if they are just placed in an environment and said, you have to go and do this, it's not going to be the same as if they are recruited to go and work in this area. They shouldn't be forced to go and work in this environment; it's a relatively new environment and something people must have time to adjust to. It's a whole shift in mindset. There are some people who never have a shift in mindset, we must come to terms with that – if you look at attitudes with regards to providing family planning, let alone termination of pregnancy, so ... but the public must not be deterred from having a TOP by any person they come into contact with, especially nurses and doctors.

## Discussion

Providers voiced a broad range of views and understandings around abortion provision and care, however, certain issues were of central concern. Providers and non-providers alike were candid about the difficulties faced by women seeking an abortion, and raised concerns about the general quality of care women received. This was underscored by a lack of post abortion contraceptive counselling viewed by providers as a missed opportunity for contraceptive initiation. Women's perceived utilisation of abortion services as a contraceptive service was largely attributed to a "break-down" in family planning services.

Reported poor contraceptive uptake amongst post abortion clients in a relatively well resourced urban area of South Africa is cause for concern. South Africa has a relatively high contraceptive prevalence (65%) compared to other sub-Saharan African countries with the Western Cape Province having better overall reproductive health care services than most other areas of South Africa [10,11].

Religious beliefs did not prevent some providers from being strong supporters of a woman's reproductive right to choose, whereas for others, it was the main reason for not being involved in abortion provision. Late gestational age abortions were particularly difficult for all providers and impacted on service provision resulting in doctors from the private sector providing the services.

In South Africa, the proportion of abortions performed in the second trimester has been consistently high since the inception of the CTOP Act. Thirty percent of abortions are performed at a gestational age of greater than 12 weeks, while in comparison, in the United States approximately 10% of abortions are performed in the second trimester [11,13]. The high rate of second trimester abortion in South Africa is a public health concern, given that every additional week of gestational age incurs a significant increase in the risk of serious complication or death [16]. Recent research in South Africa suggests health service-related barriers coupled with personal circumstances contributed to why women delayed seeking an abortion until the second trimester of pregnancy [10].

While most providers were familiar with South Africa's CTOP Act and the conditions under which a woman can request an abortion, very few engaged with the complexities and difficulties in decision-making surrounding an unplanned pregnancy and the complex reasons why women seek an abortion. Unplanned pregnancies were largely attributed to failed contraception or irresponsible sexual behaviour, yet sexual violence and non-consensual sex was rarely mentioned as possible causes of unplanned pregnancies. This is surprising given the high levels of violence against women reported in South Africa [17,18].

Of concern were the difficulties described by some providers in accessing abortion training as the shortage of abortion providers is contingent on providers opting to undergo training. Furthermore, sustaining a pool of abortion providers will be problematic if working environments are unsupportive with no incentives or recognition of the work provided.

Quality of care is not an unexpected issue to emerge and has been reported elsewhere in South Africa within the context of reproductive health services in the public health sector [7,10,19,20]. This situation underscores the need to destigmatise the issues around abortion, for clients and providers alike.

Opportunities for values clarification training designed to promote more tolerant attitudes by service providers should be expanded on and extended to health care providers working within all spheres of reproductive health care. Work undertaken by international NGOs and partners in the years after the implementation of the CTOP Act around values clarification should be continued and sustained. Such interventions have played an important role in improving the quality and continuity of care, as well as the long term health outcomes of women seeking an abortion [21].

The training and certification of registered midwives was identified as a critical step toward making high quality abortion services accessible to all women. With the recent (2004) promulgation of the CTOP Amendment Act (no.38) providing for registered nurses to be allowed to perform first trimester abortions, the pool of certified abortion providers is expected to expand. Unless issues raised by this study are addressed, it is unlikely that the Amendment Act will have the desired effect of significantly increasing the pool of providers and thereby access to safe, early abortion services.

## Conclusion

This is the first known qualitative study undertaken in South Africa exploring providers' attitudes towards abortion and adds to the body of information addressing the barriers to safe abortion services.

Several factors seemed to be at play among service providers, in terms of influencing their decisions to become involved in some way with abortion service provision. These included a combination of circumstance and personal interest. For non-providers, religious and moral beliefs and fears of being ostracised played an important role in decisions not to be involved in abortion provision. However, despite misgivings about being involved in abortion provision, non-providers were concerned about the numerous difficulties women in South Africa faced in seeking an abortion and the need for improved contraceptive provision and counselling.

Providers' reluctance to be involved in different aspects of abortion provision led to complex and fragmented levels of service provision throughout many of the health care facilities. Related to this was the need expressed by many providers for "special or dedicated abortion clinics" where people feel "committed and passionate" about what they do and thus creating a more supportive environment for both clients and providers and could contribute to sustaining a pool of abortion care providers.

There was a general lack of understanding concerning the circumstances in which health care providers were entitled to invoke their right to refuse to provide, or even assist in abortion services. Health services seemed to lack standardised structures to deal with conscientious objection among health care providers. Furthermore, there seemed to be very little recognition or support from health service managers regarding effects of conscientious objection on service provision.

### Limitations

This study was conducted in a research setting with many more designated abortion facilities than in other areas of South Africa. However, due to the country wide shortage of health care personnel, including abortion care providers, this study would have been logistically difficult to be undertaken further a field. Difficulties in accessing abortion care providers or those willing to be interviewed would have been even more difficult in areas with few abortion services and hence abortion providers.

### Recommendations

Recommendations suggested draw on a number of issues that were highlighted by providers interviewed for this study.

Contraceptive counselling, including post abortion contraceptive counselling, needs to be strengthened and integrated into post abortion care. Examples from other developing country settings, albeit in situations where abortions are restricted, have suggested that comprehensive post abortion contraceptive counselling and method choice increases post abortion contraceptive adherence. Women are likely to accept and use contraception when the service is offered as an integrated part of post abortion care [22-24].

An emphasis on quality of care is needed and would encompass all aspects of abortion provision and care including improvements in allocated space and infrastructure. Similarly, the psychosocial needs of providers must be addressed as counselling and support is required for both providers and clients.

Knowledge and understandings of the 1996 CTOP Act, including conscientious objection, needs to be strengthened amongst all health care providers including health managers.

Access to training and further opportunities for health care providers to attend values clarification workshops and abortion training needs to be encouraged and strengthened. The South African Nursing Council should consider incorporating abortion training into the nursing curriculum.

Support programmes which attract prospective abortion care providers, and retain existing providers, need to be developed and financial compensation for abortion providers needs to be considered. Currently abortion provision for mid-level providers is not recognised as a specialised or scarce skill and recognition in monetary terms for a specialised skill should be considered. This in turn may encourage more staff to volunteer to provide abortion services.

Clear policy guidelines need to be formulated for the management and provision of TOP services and health managers at the facility level need to be included and take an active role in this process. Finally, the feasibility of stand-alone abortion clinics needs to be considered in areas where the demand for TOP provision is high, such a setting can provide a supportive environment to both providers and clients.

## Competing interests

The authors declare that they have no competing interests.

## Authors' contributions

JH conceptualised and designed the study, oversaw data collection and conducted data analysis and drafted the manuscript.

KS assisted in data collection and analysis and reviewed the manuscript.

PO assisted in data collection and analysis and reviewed the manuscript.

All authors read and approved the final manuscript.

## Pre-publication history

The pre-publication history for this paper can be accessed here:


